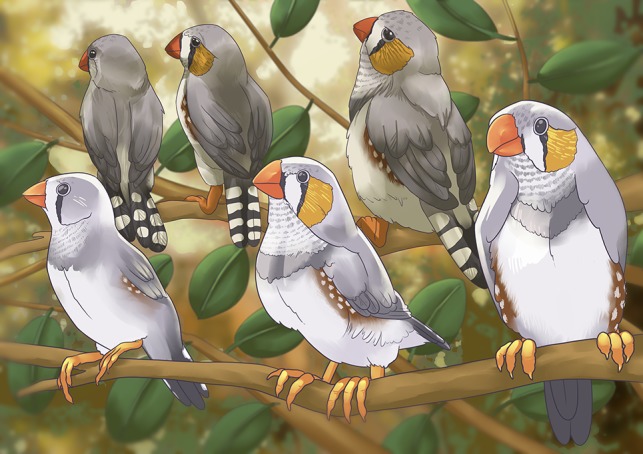# Life’s not a cinch for a stressed finch, or is it?

**DOI:** 10.1093/conphys/coy007

**Published:** 2018-02-09

**Authors:** Sofia Jain-Schlaepfer

**Affiliations:** College of Science and Engineering, ARC Centre of Excellence for Coral Reef Studies, James Cook University, 1 James Cook Dr, Douglas, QLD 4811, Australia

Why do some live longer than others? There are a number of factors in our environment that have been found to affect ageing… anything from the weather, to food availability, to disease. When there are challenges in our environment, our bodies respond to help us face these challenges. For example, when disease comes into our system, our body’s immune system takes from stored energy reserves to fight the disease. The changes in the body in response to challenges can use quite a lot of our body’s resources, resources that could instead be used for other activities. So, when we are stressed out, more of our energy may go toward overcoming what is stressing us rather than toward keeping our bodies well maintained for a long life. We might then predict that those who spend more time stressing would not live as long.

According to Marasco *et al*. (2018), the relationship between stress and ageing is, indeed, not so simple. But rather than looking at humans and the number of stressful tasks, they have to complete every day, Marasco’s team looked at zebra finches and mildly stressful reductions in food availability. They compared the survival and reproduction of birds that had as much food as they wanted to those that had reduced food availability and stress hormones added to their food during reproduction. Stressed out middle-aged finches were more likely to survive longer, while being stressed did not affect the lifespan of young or old finches. This suggests that perhaps some challenges to the body or stressful conditions can actually be beneficial when mild. Also, it seems that the life-stage at which stress occurs is an important factor too. Marasco’s team also found that finches that were stressed out at a young age were not able to reproduce as well later on; whereas, stress at an older age increased their ability to reproduce, almost like a last ditch effort to procreate.

What do these findings tell us about conservation? Conservation aims to maintain populations faced with challenges. Maintaining populations, of course, depends on the survival and reproduction of the organism that we are aiming to conserve. It is interesting that eliminating challenges will not necessarily lead to healthier individuals that reproduce better. Marasco and team also showed that it is important to consider different effects of stressors on different life stages when developing conservation practices. So, this brings to question: What are optimal stress levels? What is the degree and what are the types and numbers of challenges that will lead to the healthiest populations? What is the amount of stress in your own life that will keep you healthiest? These are the questions that Marasco’s team and other scientists will hopefully tackle next in future studies.

Illustration by Erin Walsh; Email: ewalsh.sci@gmail.com

**Figure:**